# Perceived gym service quality, leisure boredom, and loneliness among leisure sport participants: a cross-sectional study

**DOI:** 10.3389/fpsyg.2026.1871119

**Published:** 2026-10-08

**Authors:** Faik Orhun Tapşın, Gökhan Çakmak, Fuat Orkun Tapşın

**Affiliations:** 1 School of Physical Education and Sports, Dicle University, Diyarbakir, Türkiye; 2 Sports Sciences Faculty, Kocaeli University, Kocaeli, Türkiye; 3 Institute of Health Sciences Kocaeli University, Kocaeli, Türkiye

**Keywords:** gym service quality, leisure boredom, leisure time, loneliness, sports participants

## Abstract

**Aim:**

This study aims to examine the relationships among perceived gym service quality, leisure boredom, and loneliness among leisure sport participants, and to examine the statistical indirect relationship between perceived gym service quality and loneliness in relation to leisure boredom.

**Method:**

This research was conducted using a cross-sectional and correlational research design on volunteer individuals who regularly attend fitness centers in the Kayapinar district of Diyarbakir, Türkiye. Data were collected face-to-face on different days and times. In addition to a demographic information form, the Perceived Service Quality Scale, the Leisure Time Boredom Perception Scale, and the UCLA Loneliness Scale were used in the study. SPSS 26.0 software and the PROCESS Macro (v4.2) extension were used for data analysis. The data were determined to exhibit a normal distribution, and parametric tests were applied accordingly. Descriptive statistics, independent samples *t*-test, ANOVA, Pearson correlation, and hierarchical regression analyses were applied during the analysis process. Furthermore, the statistical indirect relationship between gym service quality and loneliness in relation to leisure boredom was examined using the PROCESS Macro with 5,000 bootstrap samples.

**Results:**

Correlation analysis revealed low-level, statistically significant relationships: a positive relationship between perceived gym service quality and perceived leisure boredom, a negative relationship between perceived gym service quality and loneliness, and a positive relationship between perceived leisure boredom and loneliness (*p* < 0.001). Hierarchical regression analysis identified a negative statistical relationship between gym service quality and loneliness and a positive statistical relationship between perceived leisure boredom and loneliness. The final model explained 10.8% of the total variance in loneliness scores. Analysis of statistical indirect relationships showed that while the direct statistical relationship between gym service quality and loneliness was negative, the indirect statistical relationship involving perceived leisure boredom was positive and statistically significant.

**Conclusions:**

In conclusion, negative statistical relationships were identified between perceived gym service quality and loneliness, while positive statistical relationships were found between perceived gym service quality and perceived leisure boredom, as well as between perceived leisure boredom and loneliness. Furthermore, the direct statistical relationship between gym service quality and loneliness was negative, whereas the statistical indirect relationship involving perceived leisure boredom was positive and statistically significant. Given the study's cross-sectional design and the simultaneous assessment of variables, the findings should be interpreted within the framework of the statistical relationships between the variables and the statistical decomposition of the observed cross-sectional relationships; temporal ordering cannot be established, and causal inferences cannot be drawn.

## Background

In modern societies, increasing urbanization, the integration of digital technologies into daily life, and the intensification of individuals' work schedules have significantly altered the ways in which people interact socially (Lyons et al., 2018). This transformation is associated with individuals' physical activity habits and the ways in which they spend their leisure time (Engberg et al., 2012; Hao and Cui, 2025). Today, gyms are seen not only as places where individuals improve their physical health, but also as important leisure environments where they can establish social interactions and support their psychological wellbeing (Aneja, 2025; Heckel et al., 2024). In this context, gyms are considered as versatile recreational areas that can meet both the physical and psychosocial needs of individuals (Tavares et al., 2019; Çuhadar et al., 2025).

Many studies have reported that participation in leisure sports activities is associated with positive outcomes in individuals' physical health, psychological wellbeing, social cohesion, and life satisfaction (Mutz et al., 2021; Ma and Li, 2024; Rodríguez-Bravo et al., 2020). In particular, sports environments are considered important social spaces associated with social relationships, a sense of belonging, and experiences related to coping with the stress of daily life (Walseth, 2006; Karstensen et al., 2024). However, the quality and nature of the services offered in sports environments may be associated with how individuals evaluate their experiences in these environments (Greenwell et al., 2002). The perceived quality of service in gyms is considered an important element reflecting how individuals generally evaluate their experience in the sports environment (Polyakova and Ramchandani, 2023; Dang et al., 2022). In this context, service experiences that do not adequately meet participants' expectations may be associated with less favorable evaluations of the sports environment and with differences in the psychosocial benefits associated with leisure activities.

In the context of sports services, service quality is considered an important factor in individuals' satisfaction with their sports experience and their intention to continue participating (Fernando et al., 2023). Higher perceived service quality has been linked to greater commitment to sports activities, higher satisfaction levels, and more positive experiences in sports environments (Jae Ko et al., 2011; Theodorakis et al., 2019). On the other hand, insufficient perceived service quality may be accompanied by lower motivation in the sports environment, reduced interest in activities, and greater perceptions of boredom over time (Atasayar, 2024). In particular, boredom experienced by individuals during leisure activities may occur when an activity does not meet their expectations, when environmental stimuli are insufficient, or when the experience becomes monotonous (Kara and Özdedeoglu, 2017). In the context of leisure time, boredom is considered an important psychological state that may emerge when individuals do not obtain sufficient satisfaction from the activities they participate in (Yaşartürk et al., 2017). Some studies have indicated that boredom experienced during leisure activities is accompanied by less favorable social and psychological conditions (Spruyt et al., 2018; Haller et al., 2013). In this context, boredom may also be related to feelings of loneliness (Temel and Tukel, 2021). In this context, the perceived quality of service in a fitness center may be related to how individuals evaluate their sports experience, and the perceived boredom associated with this experience may be related to the quality of leisure time experience. Considering the relationship between boredom and loneliness, leisure time boredom can be considered as a relevant variable when examining the statistical relationship between gym service quality and loneliness. This approach focuses on statistical relationships between variables based on cross-sectional data and does not imply causal interpretations.

Loneliness is defined as a subjective emotional state resulting from the fact that the quantity or quality of social relationships an individual possesses does not meet their expectations (Motta, 2021). Today, loneliness is considered a significant psychosocial problem, especially in urban life and in modern societies where individualism is increasing (Blaginin et al., 2018). Leisure time sports environments are seen as potential social spaces associated with lower levels of loneliness among individuals (Koçyigit et al., 2017; Tolukan et al., 2024). However, the failure of the services offered in these environments to meet individuals' expectations may be associated with less favorable evaluations of service quality and sports experiences among participants (Robinson, 2006). In particular, low perceived service quality may be related to the satisfaction individuals derive from their sports experiences and their experiences with sports activities. However, it should be taken into account that the direction of the relationship between service quality and perceived leisure time boredom may vary in different sports environments and participant groups. Therefore, empirical examination of this relationship is important. Examining the relationships between service quality, perceived boredom, and loneliness together may provide a better understanding of the social and psychological experiences associated with gym environments.

Although numerous studies in the literature address sports service quality, leisure experience, perceived boredom, and loneliness separately, research examining the relationships between gym service quality, perceived boredom, and loneliness within the same model is limited. This highlights the need for more comprehensive research into how the characteristics of services offered in sports settings relate to individuals' physical activity participation and psychosocial experiences. Accordingly, this study aims to examine the relationships between gym service quality and loneliness among leisure sports participants and to investigate the statistical indirect relationship between gym service quality and loneliness in relation to perceived boredom. Given the cross-sectional nature of the study, the findings are interpreted in terms of statistical relationships between the variables. The study may contribute to the sports service management literature by providing further insight into the relationships between gym service quality, perceived boredom, and loneliness, and may provide gym managers with information about variables related to participants' experiences.

**Based on the relationships discussed in the literature, the following hypotheses were formulated:**

**H1**: There is a significant negative relationship between perceived gym service quality and perceived leisure boredom.
**H2:** There is a significant positive relationship between perceived leisure boredom and loneliness.
**H3:** There is a significant negative relationship between perceived gym service quality and loneliness.
**H4:** There is a statistically significant indirect relationship between perceived gym service quality and loneliness in relation to perceived leisure boredom.

## Method

This study aims to examine the relationships among perceived gym service quality, leisure boredom, and loneliness among leisure sport participants, and to examine the statistical indirect relationship between perceived gym service quality and loneliness in relation to leisure boredom.

This quantitative research employs a descriptive, comparative, and correlational research design based on a survey model. The study has a cross-sectional design, and data on gym service quality, perceived leisure time boredom, and loneliness variables were collected from the same participants in a single data collection process. The research was conducted on individuals who attend fitness centers during their leisure time. In this context, the relationships between gym service quality, perceived leisure time boredom, and loneliness were examined, and the statistical indirect relationship between gym service quality and loneliness in relation to perceived leisure time boredom was examined. Furthermore, differences in the variables according to participants' demographic characteristics and gym usage patterns were assessed. Prior to the study, the necessary approval was obtained from the Dicle University Social and Human Sciences Ethics Committee (decision dated 02.01.2026 and numbered 1064990).

The scales used to assess gym service quality, boredom perception, and loneliness levels were employed to measure individuals' perceptions of the sports environment and their psychosocial characteristics. In this context, data obtained through surveys were evaluated to examine the relationships between gym service quality, boredom perception, and loneliness. Measuring individuals' perceptions of gym service quality, boredom levels, and loneliness levels through surveys is consistent with the study's aim of examining the relationships between the variables and the statistical indirect relationship between gym service quality and loneliness in relation to boredom perception.

### Research group

This study was conducted with adult individuals who regularly attend a total of six different fitness centers operating in the Kayapinar district of Diyarbakir. Fitness centers were included in the study based on their accessibility to adult, regular users and their willingness to permit the data collection process. No specific brand, chain, or facility size criteria were used to select the centers; instead, eligible participants who agreed to take part were recruited from the fitness centers that granted permission for data collection. A non-probability convenience sampling method was employed. Participants were recruited at the fitness centers where the study took place, and participation was entirely voluntary. The inclusion criteria were defined as being 18 years of age or older, attending the fitness center regularly, and voluntarily agreeing to participate. To determine whether fitness center usage was regular, participants were asked the question: “Do you go to the gym at least one day a week?” Individuals who answered “yes” were included in the study, while those who answered “no” were excluded. Survey data were collected from participants who agreed to take part. The collected surveys were reviewed prior to data analysis, and 25 surveys containing incomplete or invalid responses were excluded. As a result of this screening, the final sample used for analysis consisted of 409 participants. The sample size was evaluated using a power analysis performed with the G^*^Power 3.1.9.7 software, and the minimum sample size required for the planned analyses was determined to be 220. The final analysis sample of 409 participants exceeds this minimum requirement. Although the participants were recruited from six different fitness centers, facility identifiers indicating the specific center of origin were not recorded in the dataset. Consequently, participants could not be linked to specific fitness centers during the analysis phase, and potential within-facility clustering could neither be statistically assessed nor adjusted for. The sociodemographic characteristics of the participants are detailed in Table 1. Given that the study was conducted in a single district and employed a non-probability convenience sampling method, the resulting sample cannot be considered representative of all fitness center users in Türkiye. Therefore, the findings should be interpreted primarily within the context of the fitness center users in the Kayapinar district of Diyarbakir who participated in the study and the specific setting in which the research was conducted.

**Table 1:** Frequencies and percentages of demographic variables.

Demographic variables		*f*	%
Age	18–25 age	212	51.8
	26–33 age	109	26.7
	34 age and older	88	21.5
Gender	Female	146	35.7
	Male	263	64.3
Education status	High School	144	35.2
	University	235	57.5
	Postgraduate	30	7.3
Employment status	Student	163	39.9
	Employed	194	47.4
	Unemployed	52	12.7
Average session duration	0–1 h	189	46.2
	1–2 h	117	28.6
	More than 2 h	103	25.2
Gym membership duration	0–1 year	206	50.4
	1–2 years	113	27.6
	More than 2 years	90	22.0
Gym usage frequency (Per Week)	1–2 days	61	14.9
	3–4 days	236	57.7
	5 days and more	112	27.4
Gym attendance motivation	Health	83	20.3
	Weight control	69	16.9
	Bodybuilding	122	29.8
	Socialization	40	9.8
	Stress reduction	69	16.9
	Competition/Performance	26	6.4

This table shows that most participants are male, aged 18–25, university graduates, actively employed, have an average session duration of 0–1 h, a gym membership duration of 0–1 year, a gym usage frequency of 3–4 days, and their gym attendance motivation is bodybuilding.

### Data collection process and inclusion criteria

The data collection process was conducted in a total of 6 different fitness centers in the Kayapinar district of Diyarbakir province, involving individuals who participated voluntarily and used the centers at different times during weekdays and weekends. Participants were informed about the purpose of the research and the data collection process, and data were collected face-to-face on a voluntary basis. During the data collection process, the researcher first informed potential participants about the purpose, scope, and conditions of voluntary participation at the entrance or lobby area of the fitness centers. Individuals who agreed to participate completed the questionnaires independently in a designated area within the fitness center. The completed questionnaires were collected by the researcher and kept confidential. The questionnaires were administered anonymously, and no names or personally identifiable information that could directly identify participants were collected. Participants' responses were not shared with fitness center staff, and no information that would allow facility personnel to evaluate individual responses was reported. To include participants with different usage times and frequencies, data collection was carried out at different times of the day (morning, noon, and evening) and on weekdays and weekends. Data for all variables were obtained from each participant in the same data collection session and through self-report.

Pre-determined inclusion criteria were applied to select individuals to participate in the study. The primary inclusion criteria were that participants be 18 years of age or older, regularly attend fitness centers, and voluntarily agree to participate in the research. To determine regular fitness center use, the question “Do you go to the gym at least one day a week?” was added to the information form. Individuals who answered “yes” were included in the study, while those who answered “no” were excluded. Accordingly, individuals who reported attending a fitness center at least one day a week were included in the research group. The criterion of attending a fitness center at least one day a week was used to identify participants who were currently using fitness centers and could provide evaluations based on their current experiences. This criterion was considered appropriate for examining gym service quality, perceived leisure time boredom, and loneliness in the context of participants' current fitness center use.

### Data collection tools

In this study, an 8-question information questionnaire prepared by the researchers was used. This questionnaire included demographic questions such as age, gender, education level, employment status, average session duration, gym membership duration, gym usage frequency, and motivation to go to the gym. In addition to demographic questions, the Perceived Service Quality Scale, the Leisure Time Boredom Perception Scale, and the UCLA Loneliness Scale were used in this study. Calculations were made based on total scores in the analyses.

#### Perceived Service Quality Scale (SQS)

The Perceived Service Quality Scale (SQS) for Sports and Fitness Centers was developed by Uçan (2007). The scale consists of 31 items and six sub-dimensions. These sub-dimensions are: Interaction Quality (10 items), Outcome Quality (5 items), Physical Environment Quality (7 items), Exercise Equipment (3 items), Program Quality (3 items), and Environmental Conditions Quality (3 items). The scale utilizes a five-point Likert-type structure, with items scored from 1 (Strongly Disagree) to 5 (Strongly Agree). There are no reverse-scored items in the scale, and no recoding or score transformation was performed in the current study. The total SQS score was calculated by summing the scores obtained from the 31 items. Accordingly, higher total scores indicate higher perceived service quality, while lower total scores indicate lower perceived service quality. The total scores obtainable from the scale range from 31 to 155. In the scale development study, Cronbach's Alpha coefficients for the sub-dimensions were reported as follows: 0.95 for Interaction Quality, 0.92 for Outcome Quality, 0.85 for Physical Environment Quality, 0.82 for Exercise Equipment, 0.83 for Program Quality, and 0.77 for Environmental Conditions Quality. In the study conducted by Uçan (2007), the Cronbach's Alpha coefficient for the total scale score was reported as 0.95. It should be noted that independent validation studies of the SQS are limited, as the primary evidence regarding its psychometric properties is based on Uçan's (2007) doctoral dissertation. Nevertheless, the total score of the SQS was utilized for research purposes in the present study, and the Cronbach's alpha coefficient calculated based on this total score was found to be 0.89. Accordingly, higher SQS total scores indicate higher perceived gym service quality, and the direction of the correlation and regression coefficients associated with the SQS was interpreted based on this scoring approach.

#### Leisure Time Boredom Perception scale (LTBP)

The Leisure Time Boredom Perception Scale, originally developed by Iso-Ahola and Weissinger (1990), was adapted into Turkish through a validity and reliability study conducted by Kara et al. (2014). The Turkish version of the scale consists of 10 items and is scored using a 5-point Likert-type scale (1 = Strongly Disagree, 5 = Strongly Agree). The scale adapted by Kara et al. (2014) comprises two sub-dimensions: “Boredom” and “Satisfaction.” In the 10-item scale, the first five items measure the Boredom dimension, while the last five items measure the Satisfaction dimension. In the Turkish adaptation study conducted by Kara et al. (2014), no scoring procedure regarding the reverse coding of items in the Satisfaction sub-dimension was reported. Therefore, in the present study, the items were not reverse-coded, and the scoring approach reported by Kara et al. (2014) was adopted. Total scores obtainable from the scale range from 10 to 50. The Cronbach's alpha coefficients reported by Kara et al. (2014) were found to be 0.72 for the Boredom sub-scale, 0.72 for the Satisfaction sub-scale, and 0.77 for the scale as a whole. Although the two-factor structure of the scale was supported by confirmatory factor analysis in the Turkish adaptation study conducted by Kara et al. (2014), the researchers also reported the total scale score—comprising all 10 items—in addition to the Boredom and Satisfaction sub-dimensions. Furthermore, the correlations between the total scale score and both the Boredom and Satisfaction sub-dimensions were reported to be 0.83. Accordingly, the sub-scales were not evaluated separately in the present study; instead, the 10-item total scale score reported by Kara et al. (2014) was used as the basis. In the sample of this study, the Cronbach's alpha coefficient for the entire scale was calculated as 0.74.

#### UCLA Loneliness scale (UCLA)

The Loneliness Scale, developed by Russell et al. (1980), was adapted into Turkish by Demir (1989). The scale consists of 20 items and is scored using a 4-point Likert scale. Total scores obtainable from the scale range from 20 to 80, with higher scores indicating a higher level of loneliness. Items 1, 4, 5, 6, 9, 10, 15, 16, 19, and 20 are reverse-coded. After reverse-coding these items, the total scale score was calculated, and in the present study, analyses were performed based on a single-dimensional total score. The Cronbach's alpha coefficient reported by Demir (1989) is 0.96. In the present study, the Cronbach's alpha coefficient of the scale was found to be 0.91.

### Data analysis

The SPSS 26.0 program and the PROCESS Macro (v4.2) add-on developed by Hayes (2022) were used in the analysis of the data. First, normality analyses were performed and skewness-kurtosis values were examined. It was observed that the values were in the range of −1.5 to +1.5, and it was assumed that the data showed a normal distribution (Tabachnick and Fidell, 2013). Accordingly, parametric tests were used. In addition to descriptive statistics, Independent Samples *t*-Test and One-Way ANOVA were applied in the analyses. Before conducting the one-way ANOVA analyses, the homogeneity of variances was assessed using Levene's test. The assumption of homogeneity of variances was not violated. Accordingly, standard one-way ANOVA was used, and when a significant difference was found, Bonferroni-adjusted *post-hoc* comparisons were performed. Pearson correlation analysis was applied to examine the relationships between variables. The internal consistency of the measurement instruments used in the research was examined with Cronbach's Alpha reliability coefficient. Hierarchical regression analysis was conducted to examine the relationships between gym service quality, boredom perception, and loneliness. Gym service quality was entered in the first stage (Model 1), and boredom perception was added in the second stage (Model 2). The multicollinearity issue between the variables was checked by examining the VIF values. Hierarchical regression analyses were conducted after evaluating the relevant regression assumptions. The independence of errors was assessed using the Durbin–Watson statistic, and this value was determined to be 1.852 for the final model. Multicollinearity was examined using tolerance and variance inflation factor (VIF) values. The obtained values (Tolerance = 1.000; VIF = 1.000) indicated no evidence of multicollinearity. Standardized residual values ranged from −2.269 to 1.714, and no extreme standardized residual values exceeding ±3 were observed. The statistical indirect relationship between gym service quality and loneliness in relation to boredom perception was examined using PROCESS Macro's Model 4 with 5,000 bootstrap samples and a 95% confidence interval. The statistical significance of the indirect relationship was evaluated according to whether the 95% bootstrap confidence interval included zero or not. Effect size (η^2^) was also calculated to demonstrate the practical significance of the differences. Eta squared was used to assess the magnitude of the differences between groups. According to Cohen's (1988) classification, η^2^ values between 0.01 and 0.06 are considered small, values between 0.06 and 0.14 are considered medium, and values greater than 0.14 are considered large.

Due to the cross-sectional and correlational design of the study, the findings were not interpreted as causal relationships. The PROCESS Model 4 and bootstrap method were used to examine the statistical indirect relationship between the variables, and the results were not interpreted as indicating a causal mechanism.

## Results

Descriptive statistics for the scale scores are presented in Table 2, and the results of the independent samples *t*-test by gender are presented in Table 3. Table 4 presents the results of exploratory one-way ANOVA analyses examining differences in SQS, LTBP, and UCLA scores according to demographic and gym-related variables. Considering the exploratory nature of these 21 comparisons, effect sizes (η^2^) were reported to determine the magnitude of group differences, and Bonferroni-adjusted 95% confidence intervals were provided for all pairwise mean differences to account for multiple comparisons.

**Table 2:** Descriptive statistics for scales.

Scale	Min.	Max.	Mean	Std. deviation
SQS	31	155	111.84	24.786
LTBP	10	45	31.08	5.143
UCLA	21	58	43.12	9.585

For the Service Quality Scale (SQS), the minimum score was 31, the maximum score was 155, the mean was 111.84, and the standard deviation was 24.786. For the Leisure Time Boredom Perception (LTBP) Scale, the minimum score was 10, the maximum score was 45, the mean was 31.08, and the standard deviation was 5.143. For the UCLA Loneliness Scale, the minimum score was 21, the maximum score was 58, the mean was 43.12, and the standard deviation was 9.585.

**Table 3:** Independent samples *t*-test results according to gender variable (exploratory analysis).

Scale	Gender	n	*M*	SD	t	p	Cohen's d	95% CI lower	95% CI upper
SQS	Female	146	110.53	23.027	−0.798	0.425	0.084	−7.074	2.988
	Male	263	112.57	25.725					
LTBP	Female	146	30.18	4.855	−2.636	**0.009** ^*^	0.275	−2.425	−0.353
	Male	263	31.57	5.240					
UCLA	Female	146	42.94	9.967	−0.289	0.773	0.029	−2.233	1.661
	Male	263	43.22	9.384					

It was determined that there was no significant difference in Service Quality Scale (SQS) scores between female (x̄ = 110.53) and male (x̄ = 112.57) participants (*t* = −0.798, *p* = 0.425). Leisure Time Boredom Perception (LTBP) scores showed a significant difference between female (x̄ = 30.18) and male (x̄ = 31.57) participants (*t* = −2.636, *p* = 0.009). Male participants had higher LTBP scores than female participants. UCLA Loneliness Scale scores did not show a significant difference between female (x̄ = 42.94) and male (x̄ = 43.22) participants (*t* = −0.289, *p* = 0.773). Bold value and asterisk indicate statistical significance at *p* < 0.05.

**Table 4:** One-way ANOVA test results according to variables age, education level, employment status, average session duration, gym membership duration, gym use frequency, and gym attendance motivation (exploratory analysis).

		SQS						LTBP						UCLA					
Variable	*N*	*M*	SD	*F*	p	Bonferroni [95% CI]	*η^2^*	*M*	SD	*F*	p	Bonferroni [95% CI]	*η^2^*	*M*	SD	*F*	*p*	Bonferroni [95% CI]	η^2^
Age																			
18–25 age^a^	212	107.91	25.421	13.981	**< 0.001**	a-b [−8.78, 4.84] **a-c [−23.15**, −8.50]^*^b-c [−22.14, –5.58]^*^	0.064	30.43	5.111	5.775	**0.003**	a-b [−2.09,0.79] **a-c [−3.74**, −0.64]^*^ b-c [**–**3.29,0.21]	0.028	43.51	9.068	1.575	0.208	a-b [−2.86, 2.56] a-c [**–**0.93, 4.90] b-c [**–**1.16, 5.44]	0.008
26–33 age^b^	109	109.88	24.375					31.08	5.323					43.66	10.293				
34 age and older^c^	88	123.74	19.764					32.63	4.701					41.52	9.830				
Education status																			
High School^a^	144	113.20	23.010	9.324	**< 0.001**	a-b [**–**1.82, 10.54] **a-c [−27.32**, −3.88]^*^b-c [−31.28, −8.63]^*^	0.044	31.55	5.193	1.425	0.242	a-b [**–**0.47, 2.15] a-c [**–**2.63, 2.33] b-c [**–**3.38, 1.42]	0.007	43.60	9.344	0.661	0.517	a-b [**–**1.51, 3.37] a-c [**–**5.32, 3.93] b-c [**–**6.09, 2.85]	0.003
University^b^	235	108.84	25.661					30.71	5.140					42.68	9.852				
Postgraduate^c^	30	128.80	18.427					31.70	4.843					44.30	8.647				
Employment Status																			
Student^a^	163	105.47	24.255	9.491	**< 0.001**	**a-b [−16.36**, −3.95]^*^a-c [−21.56, **−2.97]** b-c [**–**11.23, 7.00]	0.045	30.37	5.045	2.702	0.068	a-b [**–**2.41,0.20] a-c [**–**3.42,0.50] b-c [**–**2.28, 1.57]	0.013	43.53	8.895	6.487	**0.002**	a-b [**–**0.63, 4.20] a-c [**–**7.05, 0.19] **b-c [−8.76**, −1.66]^*^	0.031
Employed^b^	194	115.62	25.451					31.47	5.159					41.75	10.476				
Unemployed^c^	52	117.73	19.369					31.83	5.223					46.96	6.753				
Average session duration																			
0–1 h^a^	189	101.51	25.198	37.052	**< 0.001**	**a-b [−23.46**, −10.54]^*^a-c [−28.43, −14.97]^*^ b-c [−12.12,2.72]	0.154	30.26	4.680	4.507	**0.012**	a-b [**–**2.87,0.01] **a-c [−3.11**, −0.11]^*^ b-c [**–**1.84, 1.47]	0.022	43.57	9.162	0.874	0.418	a-b [**–**1.28, 4.15] a-c [**–**2.67, 2.98] b-c [**–**1.84, 1.47]	0.004
1–2 h^b^	117	118.51	24.110					31.69	4.982					42.14	10.082				
More than 2 h^c^	103	123.21	15.709					31.87	6.098					43.42	9.872				
Gym membership duration																			
0–1 year^a^	206	109.32	24.381	6.204	**0.002**	a-b [**–**7.64, 6.13] **a-c [−17.96**, −3.09]^*^b-c[−18.09, −1.46]^*^	0.030	31.14	4.859	1.949	0.144	a-b [**–**0.70, 2.19] a-c [**–**2.23,0.88] b-c [**–**3.16,0.32]	0.010	42.93	9.339	1.022	0.361	a-b [**–**3.91, 1.48] a-c [**–**2.27, 3.55] b-c [**–**1.40, 5.11]	0.005
1–2 years^b^	113	110.07	21.338					30.39	5.314					44.14	9.105				
More than 2 years^c^	90	119.84	28.118					31.81	5.495					42.29	10.670				
Gym usage frequency (per week)																			
1–2 days^a^	61	97.89	27.389	26.915	**< 0.001**	**a-b [−19.88**, −3.76]^*^a-c [−34.98, −17.12]^*^b-c [−20.67, −7.79]^*^	0.117	29.89	3.583	5.027	**0.007**	a-b [**–**2.70,0.82] **a-c [−4.33**, −0.43]^*^b-c[−2.85, −0.04]^*^	0.024	45.79	7.526	3.612	**0.028**	a-b [**–**0.60, 5.97] a-c [0.43, 7.72]^*^ b-c[**–**1.24, 4.01]	0.017
3–4 days^b^	236	109.71	24.088					30.82	4.853					43.10	9.279				
5 days and more^c^	112	123.94	18.970					32.27	6.172					41.71	10.905				
Gym attendance motivation																			
Health^a^	83	114.80	24.928	5.119	**< 0.001**	**a-b [3.12, 26.39]** ^*^	0.060	31.19	3.980	3.809	**0.002**	**a-b** [**–**0.62, 4.25]	0.045	40.83	10.249	5.252	**< 0.001**	a-b [**–**8.95,0.04]	0.061
Weight control^b^	69	100.04	20.516					29.38	4.201					45.29	9.394				
Bodybuilding^c^	122	110.51	29.102					31.03	6.260					45.22	8.002				
Socialization^d^	40	115.75	23.079					33.63	5.480					44.05	9.301				
Stress reduction^e^	69	119.04	19.675					30.88	4.928					39.39	10.058				
Competition/ Performance^f^	26	114.85	16.171					32.04	3.117					43.31	10.158				
						a-c [**–**5.87, 14.45] a-d [**–**14.70, 12.79] a-e [**–**15.88, 7.39] a-f [**–**16.10, 16.00] b-c [**–**21.22, 0.29] **b-d [−29.90**, −1.52]^*^b-e [−31.16, −6.84]^*^ b-f [**–**31.24, 1.63] c-d [**–**18.25, 7.77] c-e [**–**19.29, 2.22] c-f [**–**19.76, 11.09] d-f [**–**12.24, 20.63]						a-c [**–**1.96, 2.28] a-d [**–**5.31,0.44] a-e [**–**2.12, 2.74] a-f [**–**4.20, 2.51] b-c [**–**3.91,0.59] **b-d [−7.22**, −1.28]^*^b-e [**–**4.05, 1.04] b-f [**–**6.10,0.77] c-d [-5.31,0.13] c-e [**–**2.10, 2.40] c-f [**–**4.23, 2.22] d-f [**–**4.59, 2.28]						**a-c [−8.32**, −0.46]^*^ a-d [**–**8.53, 2.09] a-e [**–**3.06, 5.94] a-f [**–**8.68, 3.73] b-c [**–**4.09, 4.22] b-d [**–**4.24, 6.72] b-e [1.20, 10.60]^*^ b-f [**–**4.37, 8.33] c-d [**–**3.86, 6.20] c-e [1.67, 9.99]^*^ c-f [**–**4.05, 8.87] d-f [**–**10.27, 2.43]	

^*^The asterisks (^*^) in the Bonferroni column denote statistically significant pairwise differences at the *p* < 0.05 level. Superscript letters (a–f) identify the groups compared in the Bonferroni-adjusted pairwise comparisons (e.g., a–b denotes the comparison between groups a and b); the group represented by each letter is indicated beside the corresponding category within the table. Bold values indicate statistical significance at *p* < 0.05.

In general, statistically significant differences were observed, and it was determined that the effect sizes varied. The highest effect sizes were identified for Service Quality scores according to average session duration (F = 37.052, p < 0.001, η^2^ =0.154) and for Service Quality scores according to weekly gym usage frequency (F = 26.915, p < 0.001, η^2^ = 0.117). The highest η^2^ value for LTBP scores was found to be 0.045, while it was found to be 0.061 for UCLA Loneliness Scale scores. Bonferroni-adjusted confidence intervals regarding the pairwise comparisons between groups are presented in Table 4.

The Pearson correlations among the SQS, LTBP, and UCLA scores are presented in Table 5, and the results of the hierarchical regression analysis are presented in Table 6. To examine the statistical indirect relationship between service quality (SQS) and loneliness (UCLA) in relation to leisure time boredom perception (LTBP), a bootstrapping analysis with 5,000 samples was conducted using the Hayes PROCESS Macro (Model 4). The results (Table 7) indicated a statistically significant relationship between service quality and boredom perception (Path a; B = 0.058, *p* < 0.001). A statistically significant relationship was also observed between boredom perception and loneliness (Path b; B = 0.508, *p* < 0.001). Both the total statistical relationship between service quality and loneliness (Path c; B = −0.077, *p* < 0.001) and the direct statistical relationship between service quality and loneliness after including boredom perception in the model (Path c′; B = −0.106, *p* < 0.001) were statistically significant. Importantly, the indirect statistical relationship between service quality and loneliness in relation to boredom perception (ab = 0.029) was statistically significant, as the 95% bootstrap confidence interval did not include zero (95% CI [0.016, 0.047]). This finding indicates a statistically significant indirect relationship involving boredom perception within the examined model. However, given the cross-sectional and correlational design of the study, this finding was not interpreted as evidence of a causal or mediational mechanism.

**Table 5:** Correlation analysis between scales.

Scale	Statistic	SQS	LTBP	UCLA
**SQS**	r	1		
	p			
**LTBP**	r	0.277	1	
	p	**< 0.001**		
**UCLA**	r	**–**0.199	0.196	1
	p	**< 0.001**	**< 0.001**	

A low-level positive correlation was found between the LTBP scale and the SQS scale (*r* = 0.277), a low-level negative correlation between the UCLA scale and the SQS scale (*r* = −0.199), and a low-level positive correlation between the UCLA scale and the LTBP scale (*r* = 0.196). All these correlations were statistically significant (*p* < 0.001). Bold values indicate statistical significance at *p* < 0.05.

**Table 6:** Hierarchical regression analysis of factors associated with loneliness.

Variables	Model 1			Model 2		
	B	SE B	β	B	SE B	β
(Constant)	51.724	2.152		39.214	3.055	
SQS	**–**0.077	0.019	**–**0.199^***^	**–**0.106	0.019	**–**0.274^***^
LTBP				0.508	0.091	0.272^***^
R^2^		0.040			0.108	
Adjusted R^2^		0.037			0.104	
ΔR^2^		0.040			0.068	
ΔF		16.759^***^			31.165^***^	

A hierarchical multiple regression analysis was conducted to examine the statistical relationships with Loneliness (UCLA). Preliminary analyses indicated no issues with multicollinearity, as the Variance Inflation Factor (VIF) values were within acceptable limits. In the first step (Model 1), Service Quality (SQS) was entered and accounted for a significant 4% of the variance in loneliness (ΔR^2^ = 0.040, ΔF(1, 407) = 16.759, p < 0.001). Service quality was negatively and statistically related to loneliness (β = −0.199, *p* < 0.001). In the second step (Model 2), Leisure Time Boredom Perception (LTBP) was added, which significantly improved the model by explaining an additional 6.8% of the variance in loneliness (ΔR^2^ = 0.068, ΔF(1, 406) = 31.165, *p* < 0.001). The final model (Model 2) accounted for 10.8% of the total variance in loneliness. The results of the final model demonstrated that service quality was negatively statistically related to loneliness (β = −0.274, *p* < 0.001), whereas higher boredom perception was positively statistically related to loneliness (β = 0.272, *p* < 0.001). ^***^p < 0.001.

**Table 7:** Total, direct, and indirect statistical relationships in the PROCESS model.

Paths	Relationship Type	B	SE	*t*	*p*	95% CI
SQS → LTBP (a)	-	0.058	0.010	5.817	< 0.001	[0.038, 0.077]
LTBP → UCLA (b)	-	0.508	0.091	5.583	< 0.001	[0.329, 0.686]
SQS → UCLA (c)	Total Statistical Relationship	**–**0.077	0.019	**–**4.094	< 0.001	[**–**0.114, **–**0.040]
SQS → UCLA (c′)	Direct Statistical Relationship	**–**0.106	0.019	**–**5.623	< 0.001	[**–**0.143, **–**0.069]
SQS → LTBP → UCLA (ab)	Indirect Statistical Relationship	0.029	0.008	-	-	[0.016, 0.047]^*^

Bootstrap confidence intervals (5,000 samples). ^*^The indirect statistical relationship is statistically significant because the 95% bootstrap confidence interval does not include zero.

### Testing the hypotheses

The findings showed that hypothesis H1, which proposed a significant negative relationship between perceived gym service quality and perceived boredom during leisure time, was not supported. Contrary to the direction hypothesized in H1, perceived gym service quality was positively and statistically significantly related to perceived boredom during leisure time (B = 0.058, SE = 0.010, *t* = 5.817, *p* < 0.001, 95% CI [0.038, 0.077]). Hypothesis H2, which proposed a significant positive relationship between perceived boredom during leisure time and loneliness, was supported. Perceived boredom during leisure time was positively and statistically significantly related to loneliness (B = 0.508, SE = 0.091, t = 5.583, *p* < 0.001, 95% CI [0.329, 0.686]). Hypothesis H3, which proposed a significant negative relationship between perceived gym service quality and loneliness, was supported. The total statistical relationship between perceived gym service quality and loneliness was negative and statistically significant (B = −0.077, SE = 0.019, *t* = −4.094, *p* < 0.001, 95% CI [−0.114, −0.040]). Hypothesis H4, which proposed a statistically significant indirect relationship between perceived gym service quality and loneliness in relation to perceived boredom during leisure time, was supported. The indirect statistical relationship was 0.029, with a 95% bootstrap confidence interval of [0.016, 0.047], which did not include zero. Overall, H2, H3, and H4 were supported, whereas H1 was not supported. Contrary to the negative relationship hypothesized in H1, the statistical relationship between perceived gym service quality and perceived boredom during leisure time was positive.

## Discussion

In this study, the findings obtained according to the gender variable showed that there was no significant difference between female and male participants in Service Quality Scale (SQS) scores. The literature states that the perception of service quality is related to tangible and intangible aspects of the service, such as facility amenities, staff behavior, and accessibility, and that these evaluations are shaped by experiences (Tsitskari et al., 2006; Cho, 2024). When UCLA Loneliness Scale scores were examined, no significant difference was found according to gender. Indeed, it is stated that loneliness is related to the quality of an individual's social relationships, and the relationship between gender and loneliness has not been consistently established in the literature (Kraus et al., 1993; Ekinci et al., 2015; Duru, 2008). However, a significant difference was found in Leisure Time Boredom Perception (LTBP) scores, with male participants reporting higher scores. Leisure time boredom perception is stated to be related to the levels of satisfaction and arousal individuals obtain from their leisure activities (Aydin et al., 2019). The theoretical framework developed by Ragheb and Merydith (2001) suggests a relationship between individuals' levels of participation in engaging and meaningful activities and their perception of boredom. Similarly, Weybright et al. (2015) indicated that insufficient arousal in the motivational processes of leisure behaviors may be related to the perception of boredom. The finding that only Leisure Time Boredom Perception (LTBP) scores differed significantly according to gender suggests that gender may be associated with differences in leisure time boredom perception in this sample.

When the findings were examined according to the education level of individuals attending the gym, a significant difference was found between the groups in Service Quality Scale (SQS) scores, while no significant difference was found in Leisure Time Boredom Perception (LTBP) and UCLA Loneliness Scale scores. According to the Bonferroni test results, it was determined that the difference in SQS scores was between the postgraduate group and the high school and university groups, with higher SQS scores in the postgraduate group. The difference in SQS scores according to education level can be evaluated together with the literature information that service quality evaluations may be related to individuals' previous experiences, knowledge levels, and expectations (Yu et al., 2014; Sahintürk and Atasoy, 2024). However, in this study, no significant difference was found in LTBP and UCLA scores according to education level. Factors supporting gym participation include exercises suitable to individuals' interests, group activities, and socialization opportunities (Nystrom et al., 2012; Rodrigues et al., 2019). The finding of significant differences in Service Quality Scale (SQS) scores based on education level suggests that education level may be associated with differences in perceived service quality in this sample.

In the analyses conducted according to employment status, a significant difference was found between the groups in Service Quality Scale (SQS) scores. Students' SQS scores were found to be lower than those of employed and non-employed participants. The evaluation of gym services may be related to individuals' living conditions, economic circumstances, and the ways in which they use their leisure time (Salvador et al., 2009; Giles-Corti and Donovan, 2002). In addition, service quality perceptions may also be related to individuals' previous experiences and personal evaluation standards (Hamer, 2006; Kelley and Turley, 2001). However, no significant difference was found in Leisure Time Boredom Perception (LTBP) scores according to employment status. The provision of services in fitness centers within the framework of specific programs and practices may be related to participants in different employment groups having similar service experiences (Riseth et al., 2019). In addition, boredom perception in leisure activities has been reported to be related to individuals' perceived control over the activity and the personal meaning they attribute to the activity (Bedir, 2023). A significant difference was found in UCLA Loneliness Scale scores according to employment status. In particular, non-employed participants had higher loneliness scores than employed participants. Loneliness has been reported to be related to the perceived adequacy of individuals' social relationships and their perceptions of social roles (Matook et al., 2015). In addition, perceived lack of social support and social isolation may be related to loneliness levels (Wang et al., 2018; Tsai and Reis, 2009). The finding of significant differences in UCLA scores according to employment status suggests that loneliness scores may differ across employment status groups in this sample.

When the findings were examined according to average session duration, a significant difference was found between the groups in terms of perceived service quality (SQS). According to the Bonferroni test results, this difference was found between individuals exercising for 0–1 h and those exercising for 1–2 h and more than 2 h, with higher SQS scores observed among individuals exercising for longer periods. In the service quality literature, it is stated that there may be a positive relationship between the duration of an individual's contact with the service and perceived service quality (Sun and Pan, 2023). Similarly, it is emphasized that sufficient experience duration is important in the holistic evaluation of the physical environment, interaction, and outcome dimensions of the service (Sevilmiş et al., 2024). A significant difference was also found between the groups in terms of perceived leisure time boredom (LTBP). This difference was found between individuals exercising for 0–1 h and those exercising for more than 2 h, with higher levels of boredom observed among individuals exercising for longer periods. In the context of fitness centers, a relationship has been reported between the time spent at the facility and experiences such as equipment use, program variety, and interaction with the environment (Macintosh and Doherty, 2007). Furthermore, a relationship has been reported between the variety of services offered in fitness centers, duration of participation, and individuals‘ interest in the activity (Jang and Choi, 2018). Accordingly, average session duration can be considered in relation to individuals' perceptions of leisure time boredom. However, no significant difference was found between the groups in terms of loneliness level (UCLA). It has been stated that loneliness is related more to the quality than the quantity of an individual's social relationships (Russell et al., 2012). The absence of a significant difference in loneliness according to average session duration may be considered in the context that physical presence in sports environments does not necessarily correspond to the quality or extent of social interaction. Individual exercise preferences and limited social interaction in fitness centers may also be relevant contextual factors when considering loneliness, although these factors were not directly measured in the present study.

When the findings are examined according to the gym membership duration variable, it is seen that there is a significant difference between the groups in terms of perceived service quality (SQS). According to the Bonferroni test results, this difference stems from the differences between individuals who have been members for more than 2 years and those who have been members for 0–1 year and 1–2 years, and the difference is determined to be in favor of individuals who have been members for more than 2 years. Studies in the field of gyms indicate that the duration of contact with the service is related to individuals' perceived service quality and satisfaction levels (Pradeep et al., 2020; Alexandris et al., 2004). Similarly, it is emphasized that long-term members in fitness centers have a higher perception of service quality, and this is related to customer loyalty (Huang and Kim, 2023). Accordingly, it is possible to evaluate the relationship between membership duration and individuals' ways of evaluating the service and their perceived service quality levels together. When examined in terms of the perception of boredom during leisure time, it was determined that there was no statistically significant difference between the groups according to membership duration. Studies on this subject indicate that monotonous exercise programs in sports centers may be related to boredom levels in individuals (Wolff et al., 2021), while varied and interactive programs may be inversely related to the perception of boredom (Eraslan, 2024). Accordingly, the lack of a significant difference between membership duration and perceived boredom in this study can be considered in conjunction with factors such as the diversity of the sports experience and the structure of the programs.

When the findings were examined according to the frequency of gym use, a significant difference was found between the groups in terms of perceived quality of service (SQS) in fitness centers. According to the Bonferroni test results, this difference was found between individuals who used the gym 1–2 days a week and those who used it 3–4 days and 5 days or more, and also between those who used it 3–4 days and those who used it 5 days or more, with the difference favoring individuals who attended the gym more frequently. In the sports services literature, it is stated that service experience is shaped by continuity and repetition, and that this may be related to perceived quality of service (Javadein et al., 2008). Furthermore, it is suggested that more frequent experiences of elements such as equipment use, physical environment conditions, and staff interaction in gyms may be related to individuals' evaluations of the service (Gray et al., 2015). A significant difference was also found between the groups in terms of perceived leisure time boredom (LTBP) according to the frequency of fitness center use. This difference was found between individuals who went to the gym 1–2 days a week and those who went 5 days or more, and between those who went 3–4 days a week and those who went 5 days or more. The findings show that higher levels of boredom were observed in individuals who used the gym more frequently. However, significant differences were also found between the groups in the findings regarding loneliness levels (UCLA). This difference was found between individuals who exercised 1–2 days a week and those who exercised 5 days or more, and it was determined that individuals who exercised more frequently had lower levels of loneliness. Indeed, it is stated that elements such as group exercises, shared spaces, and instructor-individual interaction may be related to the sense of social belonging in sports environments (Riseth et al., 2019; Heinrich et al., 2022). However, it is thought that the difference in loneliness levels should be evaluated not only with the frequency of use but also with the quality of social relationships established during this process.

When the findings were examined according to the variable of motivation to go to the gym, a significant difference was found between the groups in terms of perceived quality of service (SQS). According to the Bonferroni test results, this difference stemmed from the differences between individuals exercising for weight control and those exercising for health, socialization, and stress reduction purposes, and it was determined that individuals exercising for health, socialization, and stress reduction purposes had higher perceptions of service quality. It is stated that individuals participating with wellbeing-oriented motivations such as health and stress reduction can evaluate the services offered in fitness centers in a more holistic way. It is stated that this situation can be evaluated together with perceived service quality (García-Pascual et al., 2020; Yiapanas, 2025). A significant difference was also found in terms of perceived boredom during leisure time according to motivation to go to the gym. In particular, it was determined that individuals exercising for socialization purposes had higher levels of boredom than those exercising for weight control. Indeed, in the leisure literature, it is stated that the mismatch between individuals‘ expectations from an activity and their experience during the activity may be related to the perception of boredom (Yaşartürk, 2016; Kara and Gürbüz, 2022). Furthermore, it is suggested that the variety of programs and individualized exercise applications in fitness centers may be related to individuals' perception of the activity as more engaging (Maguire, 2001; Riseth et al., 2022). Accordingly, it can be considered that the social interaction experiences of individuals who participate in fitness centers for socialization purposes can be examined together with their levels of boredom and loneliness.

A positive and statistically significant relationship was found between service quality and the perception of boredom during leisure time. The direct statistical relationship between service quality and loneliness was negative and statistically significant. In contrast, the indirect statistical relationship between service quality and loneliness in relation to perceived boredom was positive and statistically significant. The opposite directions of the direct and indirect statistical relationships indicate different directions of association within the statistical model. In other words, while service quality shows a negative direct statistical relationship with loneliness, a positive indirect statistical relationship is observed in relation to perceived boredom. The literature indicates that service quality is related to user experience and psychosocial outcomes (Fernando et al., 2023). Service quality is considered a multidimensional construct encompassing elements such as the physical environment, staff interaction, and the reliability of the service provided, and is stated to be related to individuals' service experience and their perceptions of the environment (Kim and Kim, 2024). It is stated that these factors may be related to how users evaluate their sports experiences (Mohd Aznan et al., 2024). It is also noted that perceived service quality in fitness centers is related to individuals' levels of satisfaction and commitment to the environment (Karakoç et al., 2022). In this context, considering the statistical relationships between service quality, perceived boredom during leisure time, and loneliness together may contribute to understanding the psychosocial dimensions of experiences in sports environments.

In the first stage (Model 1), a negative and significant relationship was determined between the perceived quality of service included in the model and the level of loneliness. Service quality is considered a construct that includes not only physical facilities but also the individual's feeling of comfort, safety, and value in their environment (Lee and Kim, 2014). Furthermore, it is suggested that staff support, environmental quality, and opportunities for social interaction in fitness centers may be related to individuals spending more time in these environments and developing social connections (Csorba et al., 2025). Accordingly, perceived service quality in fitness centers may be linked to individuals' commitment to their environment and opportunities for social interaction, and these factors may also be considered in relation to loneliness levels.

In the second stage (Model 2), a positive and significant relationship was determined between the perception of leisure boredom included in the model and the level of loneliness. It is stated that the perception of leisure boredom is related to the individual's evaluation of the activity as insufficient, monotonous, or unsatisfying (Kara and Gücal, 2016). Accordingly, the perception of the exercise experience in fitness centers as insufficiently interesting or satisfying may be considered in relation to individuals' experiences of participation and social interaction. Indeed, it is stated that a decrease in satisfaction obtained from leisure experiences may be related to the individual's commitment to the activity (Tonietto et al., 2021).

In this study, a significant negative relationship was found between perceived gym service quality and loneliness, while a significant positive relationship was identified between perceived gym service quality and the perception of leisure boredom. It was also determined that the perception of leisure boredom is positively associated with loneliness. Furthermore, it was observed that the direct statistical relationship between perceived gym service quality and loneliness was negative, whereas the statistical indirect relationship in relation to the perception of leisure boredom was positive. Previous research also indicates that service quality in fitness centers is linked to individuals' service experiences and their evaluations of the facility. Studies conducted with fitness center users in Türkiye have reported that service quality is evaluated across various dimensions—such as staff, programs, facilities, and the physical environment—and is associated with positive service outcomes like customer satisfaction and experiential quality (Yildiz and Tüfekçi, 2010; Soygüden et al., 2015; Yildiz et al., 2018). Additionally, it has been noted that different dimensions of service quality in fitness centers are related to consumer satisfaction, trust, and behavioral intentions (Kim and Kim, 2024). Moreover, the relationship between perceived leisure boredom and loneliness has previously been examined directly, with findings indicating a link between these two variables (Ayhan et al., 2021). Accordingly, the findings of the present study suggest that the negative relationship between perceived gym service quality and loneliness should be considered in conjunction with the positive relationship between the perception of leisure boredom and loneliness.

However, the directions of the direct and statistical indirect relationships require particularly careful consideration. While the direct statistical relationship between perceived gym service quality and loneliness is negative, the statistical indirect relationship in relation to the perception of leisure boredom is positive. Consequently, the direction of the statistical indirect relationship is opposite to that of the direct statistical relationship. This finding should not be interpreted to mean that the perception of leisure boredom causally explains the negative relationship between service quality and loneliness or constitutes a causal mechanism within that relationship. Similarly, these relationships in opposite directions should not be viewed as a simple explanation of the observed link between service quality and loneliness by the perception of leisure boredom. Due to the study's cross-sectional design, the temporal ordering of the three variables cannot be established, and alternative directional relationships cannot be ruled out. For instance, it is possible that loneliness is associated with individuals' evaluations of service quality or their perceptions of leisure boredom, or that the relationship between the perception of leisure boredom and loneliness operates in a different direction. Therefore, the observed positive statistical indirect relationship should be interpreted not as an indication of a causal process or mechanism linking these variables, but rather as a statistical decomposition of the simultaneous cross-sectional associations among perceived gym service quality, the perception of leisure boredom, and loneliness. The fact that the statistical indirect relationship, while significant, is of limited magnitude also necessitates a cautious interpretation of this finding.

## Conclusion and recommendations

The research results showed a significant negative relationship between gym service quality and loneliness. Furthermore, a statistically significant indirect relationship was found between gym service quality and loneliness in relation to perceived leisure boredom. These results suggest that considering the relationships between gym service quality, leisure boredom, and loneliness together is important. However, due to the cross-sectional design of the study, these findings should not be interpreted as indicating causal relationships.

However, due to the cross-sectional design of the study, the direction and causality of the relationships between the variables cannot be determined. In particular, it cannot be ruled out that the relationships between loneliness and perceived gym service quality and perceived leisure boredom may also be inversely directed; similarly, reciprocal relationships between perceived leisure boredom and loneliness may also be possible. Therefore, the current findings do not demonstrate that the statistical relationship between gym service quality and loneliness reflects a specific causal process or that perceived leisure boredom constitutes a causal mechanism in this relationship. Consequently, alternative directional models in which loneliness is related to perceived gym service quality, as well as models in which the relationship between perceived leisure boredom and loneliness is considered in different directions, cannot be ruled out based on the current data. The findings should therefore be evaluated only within the framework of the observed relationships between the variables and the statistically indirect relationship between gym service quality and loneliness in relation to perceived leisure boredom.

In terms of demographic variables, significant differences in loneliness were found according to employment status, frequency of gym use, and gym attendance motivation. No significant differences in loneliness were found according to gender, age, education level, average session duration, and membership duration.

From an application perspective, the observed relationships among gym service quality, leisure boredom, and loneliness may provide considerations for fitness center managers when evaluating participants' experiences. However, these considerations are based on observed cross-sectional statistical relationships and should not be interpreted as evidence that changes in service quality would lead to changes in leisure boredom or loneliness. In this context, the following considerations may be relevant:

1. The variety of exercises offered in fitness centers, group activities, and practices related to participants' interest and engagement may be considered as components of the overall fitness center experience.
2. In light of the observed statistical relationship between weekly gym use frequency and loneliness, fitness centers may consider practices that support regular participation and continued engagement (Tross et al., 2024). Such practices should not, however, be interpreted as interventions demonstrated to reduce loneliness.
3. Participants' experiences regarding service quality and leisure boredom may be taken into account when evaluating the content and delivery of exercise programs, with guidance from fitness center staff.

Future research could comparatively examine the relationships between service quality, loneliness, and leisure boredom perceptions across different sports. Furthermore, using qualitative research methods to obtain more detailed information about participants' experiences in fitness centers could contribute to the literature. Additionally, longitudinal studies conducted at different time points could be used to investigate the temporal dimension of the relationships between these variables.

## Limitations

This research was conducted on individuals who regularly attend gyms in the Kayapinar district of Diyarbakir, Türkiye. Therefore, the generalizability of the findings to gym users in different regions is limited. A non-probability convenience sampling method was used in the study, and only individuals aged 18 and over were included. Consequently, the generalizability of the findings to different age groups and individuals with different sociodemographic characteristics is limited. Data were collected using a self-report survey method, which raises potential concerns regarding social desirability and recall bias. However, the fact that the data were obtained in the fitness centers used by the participants themselves and through face-to-face data collection does not completely eliminate the possibility of response bias related to social desirability and demand characteristics, especially in evaluations related to perceived gym service quality (SQS). Although the surveys were administered anonymously, no names or directly identifying information of the participants were collected, and individual responses were not shared with fitness center staff, the fact that participants were evaluating the services of the facility they used and that the data collection process was conducted within the facility does not rule out the possibility that they may have shaped their responses according to their perceived social expectations or the research context. Therefore, this potential methodological and context-based response bias should be considered, especially when interpreting the relationships between SQS and other variables. In future research, it is recommended that data be collected in settings outside the facility or through fully independent and anonymous online surveys, and that different data sources be utilized whenever possible. In this study, only the variables of gym service quality, perceived leisure boredom, and loneliness were examined; other psychosocial variables such as social support, types of motivation (Nam et al., 2023), and psychological wellbeing were not included. In addition, although the weekly frequency of gym use and average session duration of the participants were recorded, their relationships with exercise intensity and total exercise duration were not examined in detail. Finally, the region where the research was conducted has its own unique environmental and cultural characteristics regarding gym use. Therefore, caution is needed when comparing results obtained in different geographical or cultural contexts with the current findings. In light of these limitations, future research in different regions and with larger samples could utilize longitudinal designs to examine the temporal relationships and potential causal links between variables, while also including additional psychosocial variables.

The cross-sectional design of the study does not allow for the determination of the temporal ordering or causality of the relationships between the variables. Therefore, alternative directional explanations for the relationships between gym service quality, perceived leisure boredom, and loneliness cannot be ruled out based on the available data. For example, it is possible that loneliness is related to perceived gym service quality and perceived leisure boredom, or that the relationship between loneliness and perceived gym service quality is directed in the opposite direction. Similarly, a reciprocal relationship between perceived leisure boredom and loneliness cannot be ruled out. Therefore, the statistical indirect relationship identified in the cross-sectional data cannot be interpreted as representing a temporal or causal process. Furthermore, the positive statistical indirect relationship identified in relation to perceived leisure boredom is in the opposite direction to the observed negative direct statistical relationship between perceived gym service quality and loneliness. Therefore, this indirect relationship should not be interpreted as indicating that perceived leisure boredom causally explains the relationship between gym service quality and loneliness. Instead, the findings should be evaluated as a statistical decomposition of the observed cross-sectional relationships among gym service quality, perceived leisure boredom, and loneliness. The current findings should therefore be evaluated only within the framework of the observed relationships among the variables and the statistical indirect relationship between gym service quality and loneliness in relation to perceived leisure boredom. In future research, alternative directional models and the temporal ordering of these variables should be investigated using longitudinal or temporally structured research designs.

Although data were collected from a total of six different fitness centers in the study, the facility ID indicating which fitness center each participant came from was not recorded in the dataset during the data collection phase. Therefore, it was not possible to retrospectively determine the distribution of participants across fitness centers or to evaluate the effect of facility-level clustering on SQS, LTBP, and UCLA scores using ICC. Consequently, potential within-facility dependencies could not be directly controlled in the current analyses. This is particularly important for the perceived gym service quality variable, as service quality perception may be related not only to individual assessments but also to common physical, managerial, and service characteristics within the same facility. Therefore, caution is needed when interpreting the relationships between SQS and other variables, particularly the estimated SQS coefficient. In future studies, it is recommended that participants' fitness center IDs be recorded during the data collection phase, the number of participants per facility be reported, and facility-level clustering be evaluated using appropriate multilevel or clustered robust analyses.

Another limitation is that not all potential confounding variables were measured in the study. Demographic and gym-use variables were not included in the primary regression and PROCESS models, which focused on the theoretically specified relationships among the study variables. However, variables such as income and socioeconomic status, mental health, level of social support, facility and service characteristics of fitness centers, similarities and differences across fitness centers, and the quality of social relationships developed at fitness centers were not measured within the scope of the study. Therefore, some of the observed relationships between gym service quality, perceived leisure boredom, and loneliness may be related to individual, social, or facility-level factors that were not measured in the study.

## Data Availability

The raw data supporting the conclusions of this article will be made available by the authors, without undue reservation.
